# Do Likewise

**Published:** 1863-02

**Authors:** 


### DO LIKEWISE.

The *Watchman and Reflector*, of Boston, is one of the most faithful religious newspapers among our exchanges; faithful in its duty of watching against insidious infidelities, and faithful in its boldness in conferring not with flesh and blood, in its abnegation of "policy," or what is called worldly wisdom; faithful in its independence in exposing wickedness and dangerous principles whencever they may emanate. In these things it sets an example worthy of all praise, and of instant imitation by that portion of the religious press which will commend a paper, or periodical, or book, for its good things, while its moral poisons are not spoken of. If this faithfulness is not imitated on the part of those in the look-outs on the walls and watch-towers of religion, then is the ancient glory departed, and the Church is on the wane.

Pity is it that religious papers should speak pleasant things of publications which hold up the ministers of the Gospel to ridicule, as weak-minded; to contempt, as hypocrites; to pity, as tools; to execration, as false to the highest and holiest duties of their calling. Even-handed justice demands that the good things of any noticed publication should be commended; but that its evil things, its perversions, its misinterpretations, its lax principles, and its bad or false morality, should be passed over in silence, either purposely or by inadvertence, is a faithlessness to a high trust, which all right-thinking men must deplore.

The policy of the Press is become more and more akin to that of courtiers—to try and keep on good terms with everybody and everything possessed of influence. To our brethren of the Tripod, we therefore suggest, that they be on their guard, lest they fall into the same condemnation, each inquiring for himself, "Is it I."

